# Medication Adherence and Its Associated Factors among Patients with Type 2 Diabetes Mellitus Attending Primary Health Centers of Eastern Province, Saudi Arabia

**DOI:** 10.3390/medicina59050989

**Published:** 2023-05-20

**Authors:** Aseel Awad Alsaidan, Sultan Fahad Alotaibi, Ashokkumar Thirunavukkarasu, Bashayer Farhan ALruwaili, Rami Hamdan Alharbi, Maha M. Arnous, Omar Awad Alsaidan, Abdulbaqi Sulaiman Alduraywish, Yasir Abdulrahman Alwushayh

**Affiliations:** 1 Department of Family and Community Medicine, College of Medicine, Jouf University, Sakaka, Aljouf 72388, Saudi Arabia; 2 Department of Public Health, Hafr Al-Batin Health Affairs, Al Baladiyah, Hafr Al Batin 39921, Saudi Arabia; 3 Department of Family and Community Medicine, Prince Sultan Military Medical Center, Riyadh City 12233, Saudi Arabia; 4 Department of Family and Community Medicine, Security Forces Hospital Program, Riyadh City 12625, Saudi Arabia; 5 Department of Pharmaceutics, College of Pharmacy, Jouf University, Sakaka 72341, Saudi Arabia; 6 College of Medicine, Jouf University, Sakaka 72388, Saudi Arabia

**Keywords:** medication adherence, diabetes knowledge, illness perception, Saudi Arabia, T2DM

## Abstract

Medication adherence by patients with diabetes is critical, as it plays a crucial role in individuals’ long-term health and well-being. We evaluated the medication adherence, illness perception, diabetes knowledge, and associated factors among patients with type 2 diabetes mellitus (T2DM) attending primary health centers (PHCs) in the eastern province of the Kingdom of Saudi Arabia (KSA) using a validated Arabic version of a data collection form. In order to identify the variables that are associated with medication adherence, we applied a logistic regression analysis. Furthermore, we performed the Spearman test to find the correlation between medication adherence, illness perception, and diabetes knowledge. Of the 390 studied patients, 21.5% had low medication adherence, and it was significantly associated with gender (adjusted OR (AOR) = 1.89, 95% CI = 1.27–2.73, *p* = 0.003) and duration of diabetes (AOR = 0.83, 95% CI = 0.67–0.95, *p* = 0.017). Furthermore, we found a significant positive correlation between medication adherence and illness perception (rho = 0.217, *p* = 0.007) and knowledge of diabetes and medication adherence (rho = 0.425, *p* < 0.001). We recommend improving T2DM patients’ knowledge about the importance of adherence to their medication regimen in several health education sessions at the PHCs. In addition, we recommend mixed-method medication adherence assessment surveys in different parts of the KSA.

## 1. Introduction

Medication adherence refers to the extent to which a patient properly takes and follows their medication, as prescribed by their doctor [1]. For a patient to be considered as an adherent to prescribed medication, several factors must be met: the doctor’s prescriptions must be filled, the patient must remember to take their medication at the right time and with the right dose, and the patient must follow and understand the prescription’s directions. Medication adherence is critical as it improves quality of life by controlling chronic conditions and treating temporary conditions [2]. It also plays a crucial role in individuals’ long-term health and well-being, according to the World Health Organization (WHO) [3]. Medication adherence is a key factor in managing diabetes mellitus (DM). Treating patients with DM requires that they achieve optimal glycemic control, which reduces diabetes complications and the likelihood of risk and death [4,5]. To achieve this glycemic control, a patient is predicated on the rational taking of an antidiabetic regimen. Patients who are not adhering to a recommended antidiabetic medication regimen are anticipated to suffer from suboptimal glycemic control, which drastically increases the risk of diabetic complications [6]. Therefore, good adherence to their medication is a key step in managing diabetes mellitus and achieving successful self-management by patients.

Perceptions of illness are structured ideas about a disease’s signs, symptoms, progression, controllability, and causation. It has been demonstrated that patients’ views of their illness can predict various psychological and disease-related consequences, such as depression and lack of adherence to the prescribed medications [7,8]. Perceptions of illness are mainly concerned with the anxiety and depression levels normally resulting from patients who are suffering from a chronic illness such as DM. Patients with DM usually develop depression and stress, which creates their perception of the disease and certain beliefs about the cause and controllability of the disease [9]. These perceptions normally affect patients’ medication adherence because patients may perceive the cause of the disease, such as DM, as different from what the doctor prescribed. Therefore, this may force patients to not follow the prescription of the doctor. In treating these chronic diseases, more so for DM, it is, therefore, essential to assess a patient’s brief perception of the disease so that an understanding of the condition is reached to avoid the patient’s nonadherence to their medication.

Patients’ awareness of and knowledge about their chronic illness and its management are two of the essential components for their better understanding of the treatment protocols [10,11]. Previous studies demonstrated that in order to properly self-manage diabetes, a patient must have a thorough understanding of medications, food, exercise, home glucose monitoring, foot care, and necessary treatment changes. The assessment of diabetes-related knowledge among T2DM patients is a critical initial step from which to customize diabetes education programs and measure their efficacy [12,13,14].

Primary health care is important in nursing and managing diabetes and refers to a broad range of health care services offered by medical professionals for the community. Services include diagnosis, treatment, and support for managing a long-term health status [15]. In the Kingdom of Saudi Arabia (KSA), diabetes patients are followed at the primary health centers (PHCs) affiliated with the Ministry of Health (MOH). Therefore, a PHC is crucial in the management of diabetes because it allows medical professionals to assess and diagnose patients [15,16]. Medical professionals can also prescribe the proper treatment and support the patient in managing the illness. Nonadherence has been perceived as the primary contributor to patients with DM. According to Araya et al., medication nonadherence is a public health problem for such patients, and, therefore, medical adherence assessment practices ensure that the likelihood of a complication is minimized [17].

Rezaei et al., in their study on barriers to medication adherence in type 2 DM (T2DM) patients, found that patients’ perceptions about the disease were one of the main inhibitors to medical adherence. They argued that medical professionals should consider patients’ perceptions in promoting medication adherence [18]. Kini and Ho, in medication adherence improvement interventions, found that medical professionals use various interventions to improve medication adherence in patients with diabetes [19]. These are patient education, cognitive behavioral therapies, medication regimen management, and clinical consultation. Kleinsinger reported that the unmet challenge to medication adherence is the lack of public knowledge and awareness. They stated that to improve medication adherence in these patients, human behavioral change practices are needed to help patients adopt healthier ways of living and healthier habits [20]. Shahin and Kennedy alluded that personal and culture beliefs of patients are key contributors to medication adherence by patients with chronic illnesses [21]. A study conducted in the USA by Kirkman M et al., among a large adult cohort (more than 200,000) of patients with diabetes who were on non-insulin medications, stated that 69% of patients adhered to the medications. A logistic regression analysis of their study data found that medication adherence was significantly associated with the following independent variables: higher age, male gender, higher qualification, and high-income group [22]. In contrast, Balkhi et al. observed that a higher proportion of females had poor medication adherence (45.2% vs. 38.9%) [23]. According to Venkatesan et al., illiteracy, poor comorbid conditions, and poor satisfaction with health facilities are also key to the non-adherence of medications in diabetic patients, accounting for 45.4% of all cases [24]. Considering the high prevalence of T2DM in the KSA, it is critical to continuously assess the medication adherence among such patients. To the best of our knowledge, the research team could not find sufficient data in this context in the Hafr Al-Batin region of the KSA. To plan for the necessary awareness-raising program, it is essential to have region-specific data. Hence, we conducted this study to assess medication adherence and its associated factors among patients with T2DM attending different PHCs in Hafr Al-Batin, KSA. Furthermore, we evaluated T2DM patients’ brief perceptions of the illness, their diabetes knowledge, and its relationship with medication adherence. 

## 2. Materials and Methods

### 2.1. Study Design

The present survey was a multi-site cross-sectional study conducted from July 2022 to January 2023 among T2DM patients attending PHCs in Hafr Al-Batin, KSA. This region is situated in the Eastern Province of the KSA. There are 36 PHCs located in the region, serving approximately 300,000 people. We randomly selected 20 PHCs in the present study design. 

### 2.2. Sampling Strategy

We measured the sample size (*n*) based on the values described below. We applied 50% as the expected medication adherence (p), q = 1 − *p*, 5% margin of error (e), and 95% confidence interval (CI) to the Raosoft online sample size calculator [25]. The Raosoft online sample size calculator uses the same formula as the Cochran equation (*n* = z^2^pq/e^2^). Since there are vast differences across studies on medication adherence, we considered *p* as 50% (to obtain the maximum sample size). After considering all values, the minimum number (*n*) of patients with T2DM required to participate in the present study was 384, and we rounded it up to 390. The required patients with T2DM were recruited using a consecutive sampling technique for the current research. Using this method, we asked every fifth T2DM patient from the PHCs to participate in the study until we had the minimum necessary sample (*n* = 390). For the current research, 444 participants were invited after their follow-up visit to the PHCs. Following a focus group discussion with the doctors at the PHCs, the recruitment approach for every fifth patient was chosen to rule out the possibility of including patients from the same family with similar sociodemographic background traits. The research team contacted 444 eligible patients with T2DM during the data collection period. Of the 444 eligible patients with T2DM, 390 participants (required sample size for the present study) consented to participate in the current study (response rate 87.8%).

### 2.3. Inclusion and Exclusion Criteria

We only included patients with T2DM aged 18 years and above from the PHC settings affiliated with the MOH, the KSA, and patients who can read and write in Arabic. Additionally, patients must be on antidiabetic drugs for minimum one-month duration. We excluded patients who were unwilling to participate, patients diagnosed with mental illness or other types of diabetes, pediatric patients, and diabetes patients from private clinics or specialty hospitals. Furthermore, we excluded patients who were on insulin with or without oral antidiabetic drugs. 

### 2.4. Data Collection Procedure

We received ethical clearance from the regional ethics committee, Hafr Al-Batin Health Affairs, Kingdom of Saudi Arabia (approval no.: 083; dated: 21 June 2022). We briefed the respondents on the purposes and obtained informed consent from them to participate in the study. The research team collected data from the selected patients with T2DM using a standard and pretested Arabic data collection tool (please see the Supplementary File S1) that consisted of four sections. The first part of the tool inquired about the background characteristics of the participating diabetes patients. The second section inquired about the participants’ adherence to the prescribed anti-diabetic medication (Cronbach’s alpha (α) of the original scale—0.85) [26]. In this section, patients with T2DM responded either “yes” or “no” in the first seven questions (scores: 1 for no; 0 for yes), and question eight had 5-point Likert scale choices (scores: strongly disagree—1; disagree—0.75; neutral—0.50; agree—0.25; strongly agree—0). Based on the total score, we categorized them into low adherence (<6 of total score), medium adherence (6 and 7 of total score), and high adherence (8 of total score). In the third part, we assessed participants’ brief illness perception using the short form of the brief illness perception questionnaire (B-IPQ) [27]. The B-IPQ has been used for different age groups and wide range of illness in more than 20 languages globally. It has been proven to have good internal consistency and psychometric properties [28]. The B-IPQ is a standard and validated instrument that evaluates eight different domains of illness perception of diabetes patients through an 11-point Likert scale (scores ranged from 0 to 10). The assessed domains are consequences, timeline, personal control, treatment control, identity, concerns, understanding, and emotional representation. A higher total score demonstrates a more threatening view of diabetes, while a lower score shows a benign aspect of illness. The final section consisted of 10 questions that assessed patients’ knowledge related to diabetes (Cronbach’s alpha (α) value of the original scale—0.78) [14]. In this section, participants responded yes (score: 2), not sure (score: 1), or no (score: 0). We gave a reverse scoring for three items in the knowledge category. 

We adapted the questionnaire based on the existing literature [14,26,27] and a focused group discussion among family medicine, internal medicine, and public health experts. Initially, we made the instrument in English and then translated it into Arabic by following the standard protocols. The adapted data collection instrument was pretested (pilot study) among 30 T2DM patients in their local settings. All surveyed patients with T2DM indicated that all four sections were clear and easy to understand. During the analysis of the pilot study, we found that the participants filled in all questionnaire items, and no missing data were found. The Cronbach’s alpha (α) value for the adherence, perception illness, and diabetes knowledge sections of the Arabic data collection tool was 0.84, 0.79, and 0.88, respectively. Therefore, the research team used the same questionnaire for the main study. Furthermore, we excluded the pilot study participants from the main study.

### 2.5. Data Analysis

We executed the present study’s data analysis using the Statistical Package for Social Science (SPSS, V.23). The research team described the descriptive data of diabetes patients by the number, proportion, mean, and standard deviation (SD). We performed a logistic regression analysis to find the associated factors for adherence practice among diabetes patients. Furthermore, the total scores of medication adherence (test value = 0.924, *p* < 0.001), B-IPQ (test value = 0.898, *p* < 0.001), and diabetes knowledge (test value = 0.911, *p* < 0.001) did not meet the normality assumption assessed by Shapiro–Wilk normality assumption test. Hence, we analyzed the correlation among these scores using Spearman’s correlation test.

## 3. Results

Of the 390 analyzed T2DM patients’ data, the majority (53.3%) were male, belonged to the age group of 45 to 60 years (41.3%), worked in public sectors (47.2%), and were currently married (84.9%), and the mean ± SD of the duration of diabetes was 8.62 ± 4.5 (Table 1).

The responses related to diabetes knowledge are presented in Table 2. Of the 390 participants, the majority (81.8%) correctly answered that “consuming more sugar and other sweet dishes is a cause of diabetes”. However, less than one-third (29.5%) of the participants correctly answered regarding the importance of sleep to control their diabetes status.

The descriptive data mean ± SD of the participants’ brief perception of their illness (diabetes) are presented in Table 3. The highest score (6.74 ± 2.8) was observed for the statement “How much does your illness affect you mentally?”, followed by the participants’ perception about the recommended treatment for assisting their diabetes management (6.61 ± 2.8). The lowest score (4.67 ± 2.9) was noted for the question “how much diabetes affects your life?”.

**Figure 1:**
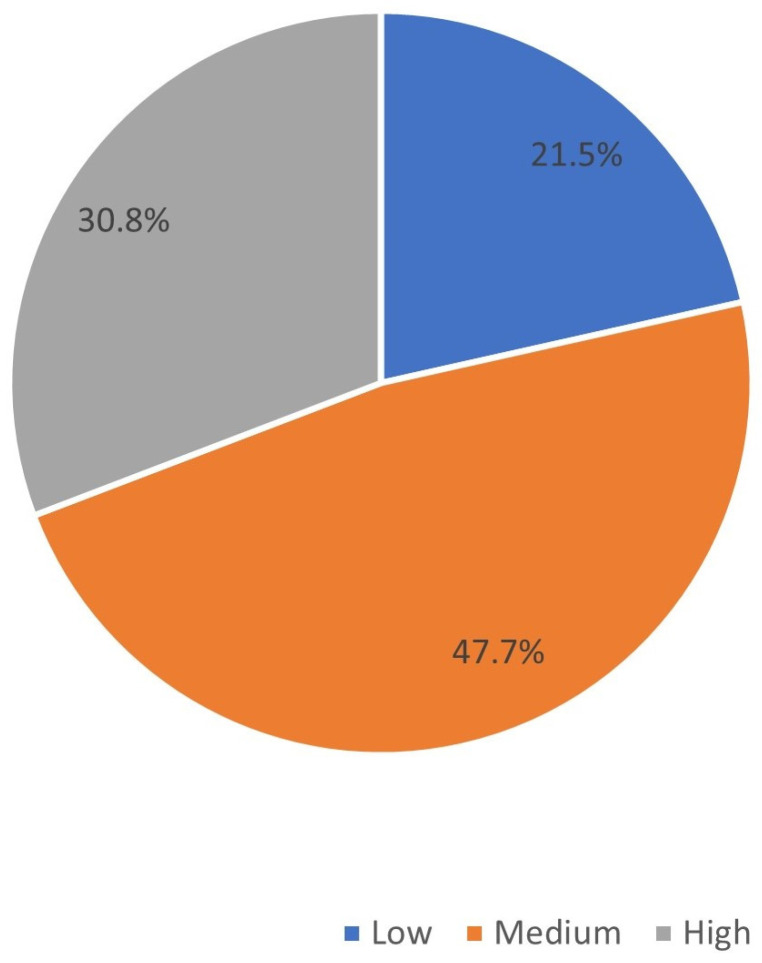
Medication adherence categories (*n* = 390).

Of the 390 samples studied, 120 (30.8%) of them belonged to the category of high-diabetes medication adherence, and 270 (69.2%) belonged to the low or medium medication adherence category (Figure 1).

Initially, we applied a binary logistic regression (low/medium vs. high) without adjusting for other variables, followed by adjusting for the other variables of our study. An unadjusted logistic regression analysis found that medication adherence was significantly associated with gender (OR = 2.43, 95% CI = 1.55–3.75, *p* < 0.001), education status (OR = 0.41, 95% CI = 0.26–0.65, *p* < 0.001), residence (OR = 1.87, 95% CI = 1.26–3.15, *p* = 0.003), monthly income (OR = 0.54, 95% CI = 0.32–0.93, *p* = 0.025), and duration of diabetes (OR = 0.76, 95% CI = 0.56–0.89, *p* = 0.003). However, after adjusting for other variables, a significant association was observed with gender (adjusted OR (AOR) = 1.79, 95% CI = 1.28–2.73, *p* = 0.003), education status (AOR = 0.65, 95% CI = 0.49–0.71, *p* = 0.001), and duration of diabetes (AOR = 0.83, 95% CI = 0.67–0.95, *p* = 0.017) (Table 4).

We found a significant positive correlation between medication adherence and B-IPQ scores (rho = 0.217, *p* = 0.007) and between diabetes knowledge and medication adherence (rho = 0.425, *p* < 0.001). No significant correlation was observed between diabetes knowledge and B-IPQ scores (Table 5).

## 4. Discussion

The present study evaluated T2DM patients’ medication adherence, knowledge, and brief perception of illness in Hafr Al-Batin, KSA. Adherence to prescribed medications by patients with diabetes is correlated with lower healthcare spending costs for a country, better clinical outcomes, decreased morbidity, decreased hospital admission rates, and decreased mortality, as stated by the Centers for Disease Control and Prevention (CDC) [29]. Diabetes education, based on a patient’s knowledge to protect their health, is the theme of the current year of the World Diabetes Day 2021–23 activities [30]. These statements from the major international health organizations restate the importance of the present study. 

The present study discovered that only 30.8% of patients with T2DM were highly adherent to the medications prescribed by doctors. A study by AlQarni et al. in AlKhobar City, KSA, reported that a slightly higher proportion (35.8%) of diabetes patients were highly adherent to medications [31]. The difference between our study and AlQarni et al.’s could be due to the inclusion of participants and study settings. Our study was conducted among T2DM patients attending PHCs, while AlQarni et al. included participants from the endocrine and diabetic clinics at a tertiary care center. An institutionally based observational survey conducted by Ayele et al. stated that a higher proportion of patients with T2DM were poorly adherent to medications [32]. Similarly, a study by Murwanashyaka et al. in 2022 also reported that a higher proportion of participants had poor adherence practices [33]. Interestingly, Balkhi et al. of the KSA reported that nearly half of their participants complied well with diabetes medication adherence [23]. These large discrepancies across these findings by different authors could be attributed to various factors, namely, study settings, tools used to assess medication adherence, and the access to and availability of appropriate diabetes care services. Furthermore, our results emphasized the need for implementing the suggested activities of the WHO and the International Diabetes Federation [30,34]. 

The present study, through a binomial logistic regression analysis, reported that the female gender was one of the significant factors associated with low medication adherence practices (AOR = 1.89, 95% CI = 1.27–2.73, *p* = 0.003). Similar to our results, Bhuyan et al. and Murwanashyaka et al. reported that females have high odds of poorly adhering to the medications prescribed by their physicians [33,35]. In contrast, in an Iranian study, a higher and more significant proportion (86.6% vs. 80.3%, *p* = 0.036) of nonadherence to diabetes medications was demonstrated among males than females, and a study from the USA by Kirkman et al. stated that good medication adherence was higher among the male sex (AOR = 1.14, 95% CI = 1.12–1.16, *p* < 0.001) [22,36]. However, a study by Chepulis et al. in New Zealand among T2DM regarding metformin medication adherence did not find a significant association between sex and medication adherence. The probable reasons for the variation across these worldwide studies could be the study settings and sociocultural variations in the different countries and regions [37]. It is worth specifying here that PHCs are divided into male and female sections in the KSA. Our study’s findings indicated that policymakers should consider targeted gender-specific programs in all PHCs.

The present study discovered that education status was one of the significant predictors of poor medication adherence among patients with T2DM. Some authors demonstrated a significant association, while others did not find a significant association between education status and medication adherence [6,22,36,38]. A recent study by Sahoo et al. from East India reported that T2DM patients with existing comorbidities and alcohol drinking habits had higher odds of having poor medication adherence [39]. Another critical predictor identified through the present survey was the duration of diabetes. Patients suffering from a longer duration of diabetes had lower odds of low medication adherence (AOR = 0.83, 95% CI = 0.67–0.95, *p* = 0.017). Our medication adherence survey findings on the duration of diabetes and medication adherence are supported by other authors [22,40]. Interestingly, a recent Chinese study discovered that patients with lower medication harm beliefs had better medication adherence toward the prescribed medications [41]. This indicates that newly diagnosed patients need special counseling sessions on the importance of adhering to the medications prescribed by their physicians and other health care workers to give better focus to newly diagnosed patients for each review. 

Even though previous studies explored several factors, adequate knowledge of diabetes and its management, including medication adherence, is essential. The present study found that participants’ diabetes knowledge is positively correlated with medication adherence (rho = 0.425, *p* < 0.001). Similar to the present study, AlShayban et al., Kassahun et al., and Haskani et al. reported that patients with a good knowledge of diabetes had better medication adherence and, in turn, better glycemic control [10,38,42]. The present study found a weakly positive correlation between participants’ brief perception of diabetes and medication adherence (rho = 0.217, *p* = 0.007). Our findings are consistent with studies by Broadbent et al. and Bilondi et al. [43,44]. Interestingly, a recent survey conducted among hypertensive Saudi patients also reported a weakly positive correlation between illness perception and medication adherence [26]. Our study’s findings and other authors’ findings indicate the importance of increasing disease knowledge among patients with T2DM. Furthermore, gaining more knowledge related to diabetes could decrease negative illness perception and increase the odds of medication adherence among patients with T2DM [12,45]. 

We executed the present medication adherence survey with the standard methodology, using a valid and reliable tool. However, the readers of the current manuscript must consider some constraints while interpreting this survey’s results.

1. We conducted this study in Hafr Al-Batin as a single-region study. Hence, the proportion of medication adherence and the predictors may not represent the total context of the KSA and the Middle East.
2. The present study did not explore the qualitative component.
3. We did not evaluate patients with T2DM who were on insulin or who had other types of diabetes.
4. The possibility of biases related to questionnaire-based cross-sectional studies, such as self-reported bias, selection bias, and recall bias, cannot be excluded from the present study’s findings.

## 5. Conclusions

The present PHC-based cross-sectional study found low medication adherence by patients with T2DM regarding their prescribed diabetes medication. Low and medium adherence were significantly associated with gender, education status, and duration of diabetes. Moreover, we found that medication adherence is positively correlated with diabetes knowledge and patients’ brief perception of their diabetes status. We recommend improving T2DM patients’ knowledge related to diabetes and note the importance of compliance with the medication regimen through several health education sessions at the PHCs and other health care facilities. These sessions can be delivered by physicians and other health care providers, namely, nurses and community pharmacists. Furthermore, we recommend mixed-method medication adherence assessment surveys in other regions of the KSA. 

## Figures and Tables

**Table 1:** Background characteristics of the participating patients with T2DM attending different primary health centers (PHCs) in Hafr Al-Batin, KSA (*n* = 390).

Variables	Frequency (*n*)	Proportion (%)
Age group		
<45 years	129	33.1
45 to 60 years	161	41.3
More than 60 years	100	25.6
Gender		
Male	208	53.3
Female	182	46.7
Employment status		
Government sector	184	47.2
Private	62	15.9
Retired	67	17.2
Unemployed	77	19.7
Education status		
University (UGs and PGs)	209	53.6
Up to high school	181	46.4
Marital status		
Married	331	84.9
Single	59	15.1
Residence		
Urban	277	71.0
Village	113	29.0
Monthly income in Saudi Riyals (SAR) (1 USD = 3.75 SAR)		
Less than 5000 (USD 1333.33)	92	23.6
5000 (USD 1333.33) to 7000 (USD 1866.67)	80	20.5
More than 7000 (USD 1866.67)	218	55.9
Smoking status:		
Yes	84	21.5
No	306	78.5
Duration of diabetes in years (mean ± SD)	8.62 ± 4.5	
Associated with other chronic disease(s)		
Yes	126	32.3
No	264	67.7

**Table 2:** Participants’ responses on the diabetes knowledge items (*n* = 390).

Knowledge Items	Yes *n* (%)	Not Sure *n* (%)	No *n* (%)
Consuming more sugar and other sweet dishes is a cause of diabetes.	319 (81.8)	37 (9.5)	34 (8.7)
If I am diabetic, my kids have a higher chance of developing diabetic.	116 (29.7)	203 (52.1)	71 (18.2)
Diabetes can be cured *.	86 (22.1)	185 (47.4)	119 (30.5)
A fasting blood sugar level of 200 is too high.	278 (71.3)	62 (15.9)	50 (12.8)
Regular exercise will increase the need for insulin or other diabetic medication *.	114 (29.2)	106 (27.2)	170 (43.6)
My diabetes status will be harder to manage, if I sleep less than 7 h per day regularly.	115 (29.5)	207 (53.1)	68 (17.4)
Cuts and abrasions on patients with uncontrolled diabetes heal more slowly.	51 (13.1)	308 (79.0)	31 (7.9)
Medication is more important than diet and exercise to control my diabetes *.	72 (18.5)	153 (39.2)	165 (42.3)
A person with diabetes should cleanse a cut with iodine and alcohol.	93 (23.8)	262 (67.2)	35 (9.0)
Diabetes can damage my kidneys.	84 (21.5)	271 (69.5)	35 (9.0)

* Reverse scoring items.

**Table 3:** Descriptive values (mean ± SD) of the brief illness perception questionnaire (B-IPQ).

Item	Mean	±SD
How much the illness (diabetes) affects your life?	4.67	2.9
What do you recognize that how long your diabetes will last?	6.58	3.2
How much is the level of control you feel over your diabetes?	5.90	2.7
What is your perception of the prescribed treatment that can help to control your illness?	6.61	2.8
How much do you suffer with the symptoms of your illness?	5.43	2.7
How much are you nervous about your illness?	5.27	2.9
How much do you perceive that you understand your disease?	5.53	2.7
How much does your illness affect you psychologically?	6.74	2.8

**Table 4:** Predictors of the medication adherence among participated patients with T2DM from PHCs in Hafr Al-Batin (*n* = 390).

Variables	Total *n* = 390	Low and Medium *n* = 270	High *n* = 120	Unadjusted Odds Ratio (95% CI)	*p* Value	Adjusted Odds Ratio (AOR) (95% CI)	*p* Value
Age group							
Less than 45 years	129	91	38	Ref		Ref	
45 to 60 years	161	106	55	1.24 (0.75–2.05)	0.394	0.97 (0.68–1.93	0.571
More than 60 years	100	73	27	0.89 (0.50–1.58)	0.683	0.64 (0.59–1.22)	0.437
Gender							
Female	182	108	74	Ref		Ref	
Male	208	162	46	2.43 (1.55–3.75)	<0.001 *	1.79 (1.28–2.73)	0.003 *
Employment status							
Government sector	184	130	54	Ref		Ref	
Private	62	54	13	1.42 (0.78–2.60)	0.256	1.68 (0.84–2.43)	0.547
Retired	67	47	30	0.58 (0.29–1.15)	0.118	0.73 (0.49–1.07)	0.097
Unemployed	77	39	23	1.54 (0.88–2.68)	0.131	1.32 (0.74–1.94)	0.108
Education status							
University (UGs and PGs)	209	127	82	Ref		Ref	
Up to high school	181	143	38	0.41 (0.26–0.65)	<0.001 *	0.65 (0.49–0.71)	0.001 *
Marital status							
Married	331	229	102	Ref		Ref	
Single	59	41	18	0.98 (0.54–1.80)	0.962	1.23 (0.71–1.74)	0.613
Residence							
Urban	277	204	73	Ref		Ref	
Village	113	66	47	1.87 (1.26–3.15)	0.003 *	1.37 (0.91–2.05)	0.067
Monthly income in Saudi Riyals (SAR) (1 USD = 3.75 SAR)							
Less than 5000	92	60	32	Ref		Ref	
5000 to 7000	80	41	39	1.78 (0.97–3.29)	0.064	1.52 (0.89–2.73)	0.113
More than 7000	218	169	49	0.54 (0.32–0.93)	0.025 *	0.73 (0.47–1.02)	0.054
Duration of diabetes (mean ± SD)	8.62 ± 4.5			0.76 (0.56–0.89)	0.003 *	0.83 (0.67–0.95)	0.017 *
Associated with other chronic disease(s)							
No	264	183	81	Ref		Ref	
Yes	126	87	39	1.01 (0.64–1.60)	0.957	1.29 (0.81–1.93)	0.838

Variable(s) adjusted in regression analysis: age group, sex, education status, employment status, marital status, residence, monthly earnings, duration of diabetes, and presence of chronic diseases. * Significant value.

**Table 5:** Spearman’s correlation among brief illness perception, diabetes knowledge, and medication adherence among participating patients with T2DM attending PHCs in Hafr Al-Batin.

	Spearman’s Rho Value	*p*-Value *
Medication adherence–B-IPQ	0.217	0.007 *
Diabetes knowledge–medication adherence	0.425	<0.001 *
Diabetes knowledge–B-IPQ	0.063	0.812

* Significant value at 0.05 level (two-tailed).

## Data Availability

The data used to analyze and produce this survey’s results will be provided by the corresponding author upon request.

## Supplementary Materials

The following supporting information can be downloaded at: https://www.mdpi.com/article/10.3390/medicina59050989/s1. File S1: Adherence to medicines among diabetes patients enrolled in various primary health centers in Hafr Al Batin, Saudi Arabia.
