# Supplementary material for: Medication Adherence and Its Associated Factors among Patients with Type 2 Diabetes Mellitus Attending Primary Health Centers of Eastern Province, Saudi Arabia
Source: Medicina (Kaunas). 2023 May 20;59(5):989. doi: 10.3390/medicina59050989 (PMC10223340; doi:10.3390/medicina59050989)
Supplement: Supplementary file 1 [file medicina-59-00989-s001.zip › medicina-2237456 - supplementary.pdf]

الالتزام بالأدوية بين مرضى السكر المسجلين بمختلف المراكز الصحية الأولية بحفر الباطن بالمملكة العربية السعودية

**البيانات الاجتماعية الديموغرافية:**	
1- العمر	
2- الجنس	1- ذكر 2- أنثي
3- محل السكن	1- حضر 2- ريف
4- المستوى التعليمي	1- أمي / يقرأ ويكتب<br>2- ابتدائي / اعدادي / ثانوي<br>3- الجامعة / الدراسات العليا
5- الوظيفة	1- موظف في قطاع حكومي<br>2- موظف في قطاع خاص<br>3- لا يعمل<br>4- متقاعد
6- الحالة الاجتماعية	1- متزوج 2- مطلق / أرمل 3- أعزب
7- الدخل الشهري	1- أقل من 5000 ريال<br>2- 5000-7000 ريال<br>3- أكثر من 7000 ريال
8- هل تدخن ؟ (بما في ذلك الشيشة)	1. نعم - يوميًا<br>2. نعم - نادرًا<br>3. لا
9- هل لديك امراض مزمنة أخري؟	1- نعم 2- لا
10-كم سنة مكتملة منذ تشخيص إصابتك بمرض السكري (حدد السنة)	
**تقييم الالتزام بالأدوية**	
1- هل تنسى أحيانًا تناول أدويتك	1. نعم<br>2. لا
2- يفقد الناس أحيانًا تناول أدويتهم لأسباب أخرى غير النسيان. بالتفكير خلال الأسبوعين الماضيين، هل كانت هناك أي أيام لم تتناول فيها أدويتك؟	1. نعم<br>2. لا
3- هل سبق لك أن توقفت عن تناول أدويتك دون إخبار طبيبك، لأنك شعرت بسوء عندما تناولتها؟	1. نعم<br>2. لا
4- عندما تسافر أو تغادر المنزل، هل تنسى أحيانًا إحضار أدويتك؟	1. نعم<br>2. لا
5- هل تناولت أدويتك أمس؟	1. نعم<br>2. لا
6- عندما تشعر بأن حالتك الصحية تحت السيطرة، هل تتوقف أحيانًا عن تناول الأدوية؟	1. نعم<br>2. لا
7- يعتبر تناول الأدوية يوميًا مصدر إزعاج حقيقي لبعض الأشخاص. هل شعرت يومًا بالإرهاق حيال الالتزام بخطة العلاج الخاصة بك؟	1. نعم<br>2. لا

8- كم مرة تجد صعوبة في تذكر تناول جميع أدويتك؟	1. أوافق بشدة<br>2. توافق<br>3. محايد<br>4. لا أوافق<br>5. أعارض بشدة
**مقياس تصور المرض :قيم تصوراتك تجاه المرض للسؤال أدناه. (0 هو الأدنى و 10 هو الأعلى)**	
1- ما مدى تأثير مرضك على حياتك؟	0 1 2 3 4 5 6 7 8 9 10
2- إلى متى تعتقد أن مرضك سيستمر؟	0 1 2 3 4 5 6 7 8 9 10
3- ما مدى سيطرة شعورك على مرضك؟	0 1 2 3 4 5 6 7 8 9 10
4- إلى أي مدى تعتقد أن علاجك يمكن أن يساعد في مرضك؟	0 1 2 3 4 5 6 7 8 9 10
5- ما مدى شعورك بأعراض مرضك؟	0 1 2 3 4 5 6 7 8 9 10
6- ما مدى قلقك بشأن مرضك؟	0 1 2 3 4 5 6 7 8 9 10
7- إلى أي مدى تشعر أنك تفهم مرضك؟	0 1 2 3 4 5 6 7 8 9 10
8- ما مدى تأثير مرضك عليك عاطفيا؟ (على سبيل المثال ، هل تجعلك غاضبًا أم خائفًا اومنزعج أو مكتئب؟	0 1 2 3 4 5 6 7 8 9 10
أسئلة معرفة مرض السكري	
1- استهلاك المزيد من السكر والأطباق الحلوة الأخرى هو سبب لمرض السكري.	1. نعم<br>2. لا<br>3. لست متأكدا
2- إذا كنت مصابًا بالسكري ، فإن أطفالي لديهم فرصة أكبر للإصابة بمرض السكري.	1. نعم<br>2. لا<br>3. لست متأكدا
3- يمكن علاج مرض السكري	1. نعم<br>2. لا<br>3. لست متأكدا
4- مستوى السكر في الدم الصائم 200 مرتفع للغاية.	1. نعم<br>2. لا<br>3. لست متأكدا
5- ستزيد ممارسة التمارين الرياضية بانتظام من الحاجة إلى الأنسولين أو أدوية السكري الأخرى	1. نعم<br>2. لا<br>3. لست متأكدا
6- سيكون من الصعب إدارة حالة مرض السكري لدي ، إذا كنت أنام أقل من 7 ساعات يوميًا بانتظام	1. نعم<br>2. لا<br>3. لست متأكدا
7- تتعافى الجروح والجروح على مرضى السكري غير المتحكم فيه بشكل أبطأ.	1. نعم<br>2. لا<br>3. لست متأكدا
8- الدواء أهم من النظام الغذائي وممارسة الرياضة للسيطرة على مرض السكري لدي	1. نعم<br>2. لا<br>3. لست متأكدا
9- يجب على الشخص المصاب بداء السكري تطهير الجرح باليود والكحول.	1. نعم<br>2. لا<br>3. لست متأكدا

10-مرض السكري يمكن أن يضر بكليتي.	1. نعم<br>2. لا<br>3. لست متأكدا

English Version of the data collection form

Adherence to medicines among diabetes patients enrolled in various primary health centers in Hafr Al Batin, Saudi Arabia

Sociodemographic data:	
1- Age (Specify completed years)	
2- Sex	1.Male<br>2. Female
3- Educational level	1. Up to high school<br>2. University / Graduate Studies
4- Occupation	1. Employee in a government sector<br>2. Employee in the private sector<br>3. Unemployed<br>4. Retired
5- Marital status	1. Married<br>2. Divorced / Widowed<br>3. Single
6- Residence	1. Rural<br>2. City
7- Monthly income	1. Less than 5000 riyals<br>2. 5000-7000 SAR<br>3. More than 7000 riyals
8- Do you smoke ? (including shisha)	1- Yes – Daily<br>2- Yes – Rarely<br>3- No
9- Do you have other chronic diseases such as hypertension, heart disease etc ?	1. Yes<br>2. No
10- How many years completed since you are diagnosed with diabetes (Specify the year)	
**Medication adherence assessment**	
1- Several times I forget to take my antidiabetic medications	1. Yes<br>2. No

2- Are you careless at times about taking your antidiabetic medication?	1. Yes<br>2. No
3- When you feel better, do you sometimes stop taking your antidiabetic medication?	1. Yes<br>2. No
4- When you travel or leave home, do you sometimes forget to bring your medicines?	1. Yes<br>2. No
5- Did you take your medications yesterday?	1. Yes<br>2. No
6- In the past month, have you did not take the prescribed doses?	1. Yes<br>2. No
7- Have you ever felt overwhelmed by sticking to your treatment plan as it might have created nuisance to you?	1. Yes<br>2. No
8- I know better than the doctors when to stop taking medication.	1. Strongly agree<br>2. Agree<br>3. Neutral<br>4. Disagree<br>5. Strongly disagree
**Disease perception** scale: Rate your perceptions towards illness for the below question. (0 is lowest and 10 is highest)	
1- How much does your illness (diabetes) affect your life?	0 1 2 3 4 5 6 7 8 9 10
2- What do you recognize that how long your diabetes will last?	0 1 2 3 4 5 6 7 8 9 10
3- How much control does your feeling take over your illness (diabetes)?	0 1 2 3 4 5 6 7 8 9 10
4- What is your perception of the prescribed treatment that can help to control your illness?	0 1 2 3 4 5 6 7 8 9 10
5- How much do you suffer with the symptoms of your illness?	0 1 2 3 4 5 6 7 8 9 10
6- How much are you nervous about your illness?	0 1 2 3 4 5 6 7 8 9 10
7- To what extent do you feel you understand your illness?	0 1 2 3 4 5 6 7 8 9 10
8- How much does your illness affect you psychologically? (For example, does it make you angry, scared, upset, or depressed?	0 1 2 3 4 5 6 7 8 9 10
**Diabetes Knowledge Questions**	

1- Consuming more sugar and other sweet dishes is a cause of diabetes.	1. Yes<br>2. No<br>3. Not sure
2- If I am diabetic, my kids have a higher chance of developing diabetic.	1. Yes<br>2. No<br>3. Not sure
3- Diabetes can be cured	1. Yes<br>2. No<br>3. Not sure
4- A fasting blood sugar level of 200 is too high.	1. Yes<br>2. No<br>3. Not sure
5- Regular exercise will increase the need for insulin or other diabetic medication	1. Yes<br>2. No<br>3. Not sure
6- My diabetes status will be harder to manage, if I sleep less than 7 hours per day regularly	1. Yes<br>2. No<br>3. Not sure
7- Cuts and abrasions on patients with uncontrolled diabetes heal more slowly.	1. Yes<br>2. No<br>3. Not sure
8- Medication is more important than diet and exercise to control my diabetes	1. Yes<br>2. No<br>3. Not sure
9- A person with diabetes should cleanse a cut with iodine and alcohol.	1. Yes<br>2. No<br>3. Not sure
10- Diabetes can damage my kidneys.	1. Yes<br>2. No<br>3. Not sure
